# CEUS in the evaluation of focal salivary gland lesions: a systematic review

**DOI:** 10.1007/s40477-026-01205-0

**Published:** 2026-07-27

**Authors:** Alessio Frisone, Giuseppe Balice, Matteo Serroni, Gianmaria D’Addazio, Antonella Berardicurti, Andrea Boccatonda

**Affiliations:** 1 Department of Innovative Technologies in Medicine and Dentistry, “G. D’Annunzio” University, Via Dei Vestini 31, Chieti, Italy; 2 Department of Life Science, Health and Health Professions, Link Campus University, Macerata, Italy; 3 Department of Medicine and Science of Aging, University Gabriele d’Annunzio Chieti-Pescara, Chieti, Italy; 4 Diagnostic and Therapeutic Interventional Ultrasound Unit, IRCCS Azienda Ospedaliero-Universitaria di Bologna, Policlinico Sant’Orsola-Malpighi, Via Massarenti N. 9, 40138 Bologna, Italy

**Keywords:** Contrast-enhanced ultrasound, Salivary gland tumors, Parotid gland, Ultrasound, Cancer

## Abstract

**Background:**

Preoperative differentiation of benign and malignant salivary gland lesions remains challenging. Contrast-enhanced ultrasound (CEUS) enables real-time assessment of tumor microvascularization and has emerged as a promising adjunct to conventional ultrasound.

**Purpose:**

To systematically review the evidence on CEUS in focal salivary gland lesions and to evaluate its diagnostic accuracy, including feature-level diagnostic performance.

**Materials and methods:**

A systematic search of PubMed was conducted for studies published from database inception to December 2025, evaluating contrast-enhanced ultrasound (CEUS) in focal salivary gland lesions, using histopathology as the reference standard. Qualitative and quantitative CEUS features were synthesized. Diagnostic accuracy evidence was assessed at both study and feature levels. Risk of bias was evaluated using the QUADAS-2 tool.

**Results:**

Ten studies comprising 663 focal salivary gland lesions (470 benign and 193 malignant) were included in the qualitative synthesis. Malignant lesions were consistently associated with heterogeneous enhancement, ill-defined or blurred margins, non-enhancing areas suggestive of necrosis, and altered contrast kinetics. Quantitative CEUS analyses demonstrated shorter time to peak enhancement, higher peak enhancement, steeper wash-in slopes, and larger areas under the time–intensity curve in malignant tumors compared with benign lesions. Only a limited number of studies reported diagnostic accuracy outcomes using predefined criteria, and substantial heterogeneity in diagnostic thresholds and study design precluded reliable pooled meta-analysis. Across the two studies reporting complete diagnostic accuracy data, CEUS sensitivity ranged from 72.1 to 80.0%, whereas specificity ranged from 78.9 to 91.7%. Feature-level analysis indicated that ill-defined enhancement margins were highly specific for malignancy, whereas inhomogeneous enhancement showed higher sensitivity but limited specificity. Wash-in and wash-out kinetics demonstrated variable diagnostic performance across studies.

**Conclusion:**

CEUS provides valuable functional information for the evaluation of focal salivary gland lesions and complements conventional ultrasound by depicting tumor microvascular characteristics. While several qualitative and quantitative CEUS features are associated with malignancy and may aid preoperative risk stratification, methodological heterogeneity and the limited number of high-quality diagnostic accuracy studies currently restrict definitive conclusions. CEUS should be considered an adjunctive imaging tool rather than a standalone alternative to histopathological diagnosis.

## Introduction

Salivary gland tumors constitute a heterogeneous group of neoplasms characterized by marked histological diversity and highly variable biological behavior. According to the World Health Organization classification, more than 30 distinct benign and malignant histotypes have been described, reflecting the biological and morphological complexity of these tumors [1]. Although the majority of salivary gland lesions are benign, malignant tumors account for a clinically relevant proportion and may present with indolent growth and nonspecific imaging features, particularly in low-grade malignancies [2]. Accurate preoperative differentiation between benign and malignant lesions is therefore crucial for appropriate surgical planning, determination of the extent of resection, prognostic stratification, and informed patient counseling.

Conventional B-mode ultrasound, often combined with color or power Doppler imaging, is widely accepted as the first-line imaging modality for the evaluation of focal salivary gland lesions [3–5]. Ultrasound offers high spatial resolution, real-time assessment, and wide availability [6]. However, despite these advantages, conventional ultrasound remains limited in its ability to reliably distinguish benign from malignant tumors, as significant overlap exists in grayscale appearance and vascular patterns [3, 7 ]. This limitation is particularly evident in tumors with complex internal architecture, cystic or necrotic components, and in low-grade malignancies that may mimic benign lesions [4, 7 ].

Contrast-enhanced ultrasound (CEUS) enables real-time visualization of microvascular perfusion through the administration of intravascular contrast agents and provides functional information beyond conventional morphological imaging [8–11]. By depicting tumor angiogenesis and perfusion dynamics, CEUS has been increasingly explored in head and neck imaging, including the evaluation of salivary gland lesions [12–14]. Several studies have reported characteristic CEUS patterns associated with malignancy, such as heterogeneous enhancement, irregular or ill-defined lesion margins, non-enhancing areas suggestive of necrosis, and rapid contrast wash-in and wash-out kinetics [12, 13, 15–17 ].

Despite growing interest and an expanding body of literature, the diagnostic performance of CEUS in salivary gland imaging remains incompletely defined. Reported results vary widely across studies, reflecting differences in study design, patient selection, CEUS acquisition protocols, contrast agent dosing, post-processing software, and interpretation criteria [14, 18, 20 –]. Moreover, many investigations focus on descriptive or quantitative perfusion patterns without defining standardized diagnostic thresholds or dichotomous decision rules, limiting the comparability of results and the feasibility of quantitative synthesis.

Against this background, a comprehensive synthesis of the available evidence is warranted. The aim of this systematic review was to summarize current knowledge on the application of CEUS in focal salivary gland lesions and to evaluate its diagnostic accuracy for differentiating benign from malignant lesions. In addition to study-level diagnostic performance, we also explored the diagnostic value of individual CEUS features to better elucidate their potential role in clinical decision-making and preoperative risk stratification.

## Materials and methods

### Study design and reporting

This systematic review was conducted in accordance with the Preferred Reporting Items for Systematic Reviews and Meta-Analyses (PRISMA) 2020 statement and the PRISMA-DTA extension for diagnostic test accuracy studies. The review protocol was defined a priori, and all methodological steps were performed following established recommendations for systematic reviews of diagnostic imaging studies.

### Eligibility criteria

Studies were considered eligible if they met all of the following criteria:

1. included adult patients with focal salivary gland lesions involving the parotid, submandibular, sublingual, or minor salivary glands;
2. evaluated contrast-enhanced ultrasound (CEUS) as the index test;
3. used histopathological examination as the reference standard;
4. reported qualitative and/or quantitative CEUS findings, with or without diagnostic accuracy data.

Exclusion criteria comprised case reports, narrative reviews, conference abstracts, non-human studies, diffuse inflammatory salivary gland diseases, and studies lacking histological confirmation of the diagnosis.

### Literature search

A comprehensive literature search was performed in PubMed from database inception to December 2025. The search strategy combined controlled vocabulary and free-text terms related to contrast-enhanced ultrasound and salivary gland pathology. Search terms included combinations of: “contrast-enhanced ultrasound,” “CEUS,” “salivary gland,” “parotid,” “submandibular,” “tumor,” and “lesion”. The full electronic search strategy was developed a priori and adapted as necessary to optimize sensitivity. No language or publication status restrictions were initially applied. In addition, the reference lists of all included studies and of relevant review articles were manually screened to identify additional potentially eligible publications that may not have been captured by the electronic search.

### Study selection and data extraction

Two reviewers independently screened titles and abstracts for potential eligibility. Full texts of potentially relevant articles were subsequently assessed for inclusion. Disagreements at any stage were resolved by consensus.

Data extraction was performed independently by both reviewers using a predefined data extraction form. Extracted variables included study design, patient demographics, lesion location, CEUS acquisition protocol, qualitative and quantitative CEUS features, reference standard, and diagnostic accuracy data (true positives, false positives, true negatives, and false negatives) when available.

### Risk of bias assessment

The methodological quality and risk of bias of the included diagnostic accuracy studies were assessed using the Quality Assessment of Diagnostic Accuracy Studies-2 (QUADAS-2) tool. Each study was independently evaluated across the four standard QUADAS-2 domains: patient selection, index test (CEUS), reference standard, and flow and timing. For each domain, risk of bias was judged as low risk, high risk, or no information according to the QUADAS-2 signaling questions. In addition, applicability concerns were assessed for the patient selection, index test, and reference standard domains and categorized as low, some concerns, or high. Discrepancies between reviewers were resolved by consensus.

### Statistical analysis

When sufficient data were available, diagnostic test accuracy meta-analysis was performed using a bivariate random-effects model to jointly estimate pooled sensitivity and specificity while accounting for the potential correlation between these two measures across studies. Between-study heterogeneity was assessed qualitatively because of the limited number of eligible studies included in the meta-analysis, precluding reliable quantitative estimation of heterogeneity parameters or threshold effects. Given the small number of included studies and the variability in diagnostic criteria and decision thresholds across investigations, pooled estimates were considered exploratory rather than definitive. Accordingly, results were interpreted with caution, and no formal assessment of publication bias or subgroup analyses was performed.

## Results

### Study selection

The PubMed search identified 1453 records. Following title and abstract screening, 67 full-text articles were assessed for eligibility. Fifty-seven studies were excluded after full-text review, leaving ten studies for inclusion in the qualitative synthesis. Studies were excluded because they did not evaluate CEUS as the index test, lacked histopathological confirmation, focused on diffuse salivary gland disease rather than focal lesions, were review articles, case reports, conference abstracts, or did not provide extractable CEUS data relevant to the objectives of the review. Ultimately, ten studies fulfilled the inclusion criteria and were included in the qualitative synthesis [12–21–24,18 ].

### Risk of bias assessment

**Fig. 1:**
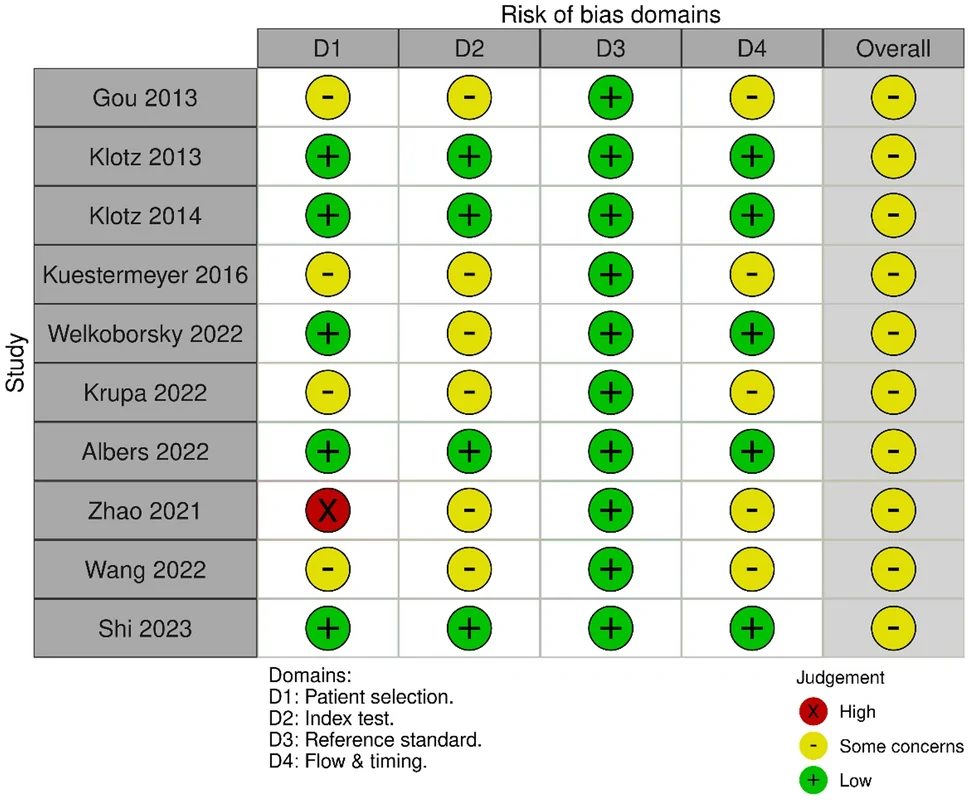
Risk of bias assessment of included studies using the QUADAS-2 tool. Each study was evaluated across the four standard QUADAS-2 domains: patient selection (D1), index test (D2), reference standard (D3), and flow and timing (D4). Judgements are shown as low risk (green), high risk (red), some concerns (yellow), or no information (blue). An overall judgement is provided for each study based on domain-level assessments

**Fig. 2:**
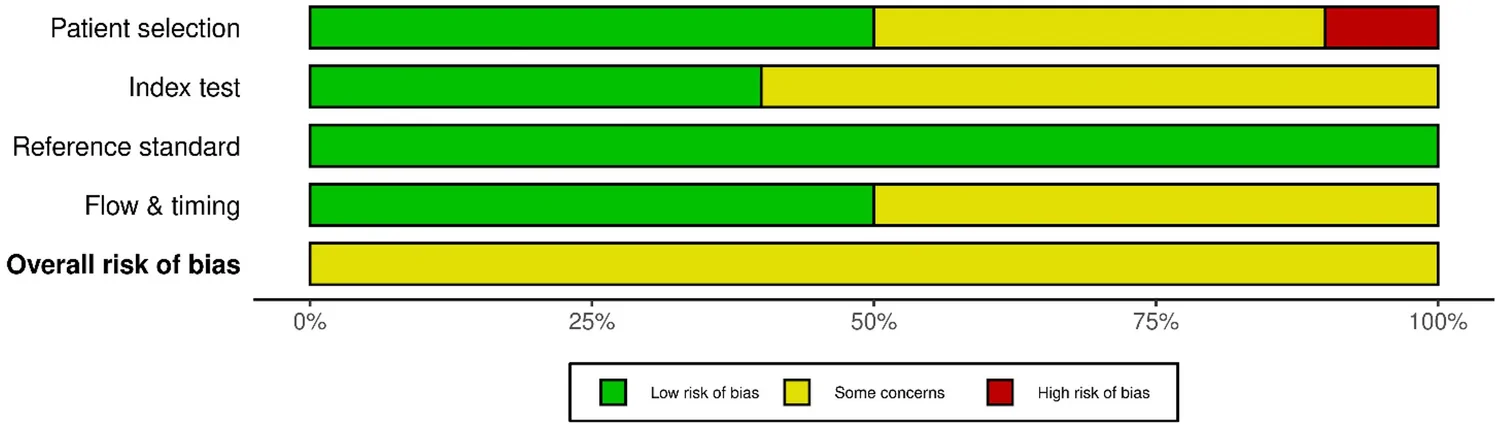
Summary of risk of bias and applicability judgments across included studies using the QUADAS-2 tool. The stacked bar chart shows the proportion of studies rated as low risk of bias (green), high risk of bias (red), some concerns (yellow), or no information (blue) for each QUADAS-2 domain: patient selection, index test, reference standard, and flow and timing, as well as the overall risk of bias

Risk of bias was assessed using the QUADAS-2 tool across the four standard domains: patient selection, index test, reference standard, and flow and timing (Figs. 1 and 2). Overall, the methodological quality of the included studies was moderate. Most investigations were judged as having a low overall risk of bias or some concerns. In the patient selection domain, a substantial proportion of studies were rated as some concerns or high risk of bias. This was mainly attributable to retrospective study design, lack of explicit reporting of consecutive or random patient recruitment, and insufficient description of inclusion and exclusion criteria, as observed in several retrospective cohorts. In some investigations, highly selected patient populations were enrolled or complex and indeterminate lesions were excluded, raising concerns about spectrum bias and limiting generalizability to routine clinical practice [13, 17, 24,18 ]. For the index test domain, risk of bias was frequently judged as some concerns, reflecting insufficient reporting of CEUS interpretation criteria, absence of predefined diagnostic thresholds, and inconsistent reporting of blinding of image readers to the reference standard [13, 17, 24,18 ]. Only a minority of prospective studies explicitly stated that CEUS images were interpreted without knowledge of histopathological results [14, 16, 23,22 ]. In addition, several studies evaluated multiple qualitative features or quantitative perfusion parameters without defining a single a priori diagnostic decision rule, increasing the risk of optimism bias and data-driven threshold selection. The reference standard domain was consistently rated as low risk of bias across almost all included studies, as histopathological examination of surgical specimens or biopsy samples was used as the reference standard. Nevertheless, explicit reporting of blinding of pathologists to CEUS findings was frequently lacking, resulting in several judgments of no information in this domain despite the intrinsic robustness of histology as a reference standard. In the flow and timing domain, most studies were judged as having either low risk of bias or some concerns. CEUS was generally performed preoperatively with subsequent histological confirmation. However, incomplete reporting of the time interval between CEUS and surgery or biopsy, as well as insufficient description of patient exclusions after enrollment or losses to follow-up, contributed to several no information ratings, particularly in retrospective studies [13, 17, 24,18 ]. Overall applicability concerns were generally low. Nevertheless, some studies raised some concerns due to the predominance of parotid gland lesions and the under-representation of inflammatory lesions, minor salivary gland tumors, and indeterminate masses [16, 19 ]. Additional applicability concerns were related to heterogeneity in CEUS acquisition protocols, contrast agent dosing, region-of-interest selection, and interpretation criteria, which may limit the generalizability of results to centers using different equipment or contrast administration techniques.

### Study characteristics

Ten studies comprising 663 salivary gland lesions (470 benign and 193 malignant) were included in the qualitative synthesis [13, 14, 16–22–25,19 ] (Table 1). Most investigations were single-center observational studies, with a predominance of retrospective designs [13, 17, 24,18 ], while a smaller number were conducted as prospective cohorts [14, 16, 25,19,22,23 ]. Sample sizes varied considerably across studies, ranging from small prospective series including approximately 25–40 lesions [19, 22, 25 ] to larger retrospective cohorts enrolling more than 90–100 patients [16–18].

**Table 1:** Main characteristics of studies included in the systematic review evaluating contrast-enhanced ultrasound (CEUS) in focal salivary gland lesions

First author (year)	Country	Study design	Salivary gland(s)	Sample size (benign/malignant)	Reference standard	Ceus approach	Quantitative analysis	Diagnostic accuracy data
Gou (2013)	China	Retrospective	Mixed (parotid, submandibular)	68 (51/17)	Surgery/biopsy	Qualitative CEUS + TIC	Yes	No
Klotz (2013)	Germany	Prospective	Parotid	39 (32/7)	Surgery	Quantitative CEUS (TIC)	Yes	No
Klotz (2014)	Germany	Prospective	Parotid	40 (32/8)	Surgery	Quantitative CEUS (TIC)	Yes	No
Küstermeyer (2016)	Germany	Prospective	Mixed	25 (21/4)	Surgery/biopsy	Qualitative CEUS + ROI perfusion	Yes	No
Welkoborsky (2022)	Germany	Prospective	Parotid	100 (92/8)	Surgery	Qualitative CEUS (heterogeneity patterns)	No	No
Krupa (2022)	Poland	Retrospective	Mixed	63 (37/26)	Surgery	Qualitative CEUS + TIC	Yes	Partial
Albers (2022)	Germany	Prospective	Parotid	58 (44/14)	Surgery	Quantitative CEUS + elastography	Yes	No
Zhao (2021)	China	Retrospective	Mixed	64 (19/45)	Surgery/biopsy	Qualitative CEUS	No	Yes
Wang (2022)	China	Retrospective	Mixed	91 (70/21)	Surgery/biopsy	Qualitative CEUS	No	No
Shi (2023)	China	Prospective	Parotid	115 (72/43)	Surgery	Qualitative CEUS ± elastography	No	Yes

The table summarizes study design, population characteristics, salivary gland involvement, reference standard, contrast-enhanced ultrasound assessment approach—including qualitative evaluation and time–intensity curve (TIC) analysis—and the availability of diagnostic test accuracy (DTA) data for malignancy. When applicable, conventional ultrasound (US) findings are also reported. NR indicates data not reported

The parotid gland was the most frequently investigated anatomical site and represented the exclusive or predominant focus in the majority of studies [14, 16, 25,19,22,23 ]. Several investigations included mixed salivary gland populations, most commonly involving both the parotid and submandibular glands [13, 17, 24,18 ]. Minor salivary gland tumors were rarely represented, limiting conclusions regarding these less common entities [13, 17, 18 ]. Across studies, benign lesions were more prevalent than malignant tumors, reflecting real-world disease distribution, with malignant lesions accounting for approximately 15–35% of included cases [13, 14, 16–24,18,23 ].

The reference standard was based on histopathological examination of surgical specimens in all included studies, with biopsy results also included in a minority of investigations [13, 17, 24,18 ]. In all cases, histology served as the final diagnostic reference for classification of lesions as benign or malignant.

Considerable heterogeneity was observed in CEUS acquisition protocols and image analysis methods. Most studies employed second-generation ultrasound contrast agents, although contrast agent type, dose, injection technique, and imaging duration varied across investigations. Region-of-interest selection, post-processing software, and perfusion parameter extraction methods also differed substantially among studies, particularly in investigations performing quantitative analysis [13, 14, 25,19,22,23 ].

Interpretation approaches ranged from purely qualitative visual pattern analysis [16–18] to quantitative time–intensity curve–based perfusion analysis [13, 14, 25,19,22,23 ], as well as combined multiparametric models integrating CEUS with additional ultrasound techniques [16, 23 ]. Qualitative CEUS assessments focused on enhancement homogeneity, lesion margins, wash-in and wash-out kinetics, and the presence of non-enhancing areas [16–24,18 ]. Quantitative analyses evaluated parameters such as time to peak, peak enhancement, wash-in slope, area under the time–intensity curve, and mean transit time [13, 14, 25,19,22 ].

Notably, diagnostic thresholds and decision rules were variably defined or not predefined at all, and only a minority of studies reported a global dichotomous CEUS classification (malignant vs benign) suitable for diagnostic accuracy meta-analysis [16, 24 ]. Overall, substantial methodological heterogeneity was present across studies with respect to patient selection, lesion spectrum, CEUS acquisition protocols, feature definitions, and interpretation criteria, limiting direct comparability across investigations and contributing to the restricted number of studies eligible for quantitative synthesis.

### Qualitative CEUS patterns

Across the included studies, malignant salivary gland lesions were consistently associated with a distinct set of qualitative CEUS features reflecting abnormal tumor microvascular architecture (Table 2). In particular, malignant tumors more frequently demonstrated heterogeneous intralesional enhancement, ill-defined or blurred enhancement margins, and the presence of non-enhancing areas suggestive of necrosis [13, 16, 24 –18 ]. These findings are biologically plausible and likely reflect tumor angiogenesis, vascular disorganization, and infiltrative growth patterns. Altered contrast dynamics were also commonly reported in malignant lesions, with several studies describing a pattern of rapid contrast wash-in followed by relatively early wash-out, although the diagnostic value of wash-in and wash-out timing alone was inconsistent across investigations [16–24,18 ]. In contrast, benign salivary gland tumors more frequently exhibited homogeneous and well-demarcated enhancement, with regular vascular architecture and absence of non-enhancing intralesional areas [13, 14, 25,19,22 ]. Notably, substantial overlap in qualitative CEUS appearance was observed between malignant tumors and certain benign or inflammatory lesions. Highly vascular benign entities, such as Warthin tumors, as well as focal inflammatory masses, frequently showed heterogeneous enhancement and increased perfusion, potentially mimicking malignant patterns [13, 18, 24 ]. This overlap limits the specificity of individual qualitative CEUS features and underscores the importance of interpreting CEUS findings within a multiparametric diagnostic framework, integrating clinical presentation and conventional ultrasound features.

**Table 2:** Qualitative contrast-enhanced ultrasound (CEUS) patterns reported across studies for benign and malignant focal salivary gland lesions

CEUS qualitative feature	Benignlesions	Malignantlesions
Enhancement homogeneity	Typically homogeneous enhancement	Frequently heterogeneous enhancement
Lesion margins on CEUS	Well-defined, regular margins	Ill-defined or blurred margins
Non-enhancing areas	Rare	Common (suggestive of necrosis or hypoperfusion)
Wash-in pattern	Gradual or moderate wash-in	Rapid wash-in
Wash-out pattern	Slow or absent wash-out	Early or rapid wash-out
Vascular architecture	Regular, organized microvasculature	Irregular, disorganized vascular pattern

The table summarizes the most frequently described CEUS features and their typical association with lesion histology

### Quantitative CEUS parameters

Quantitative CEUS analyses across the included studies demonstrated systematic differences in perfusion-related parameters between malignant and benign salivary gland lesions, although with substantial inter-study variability (Table 3). Malignant tumors generally exhibited shorter time-to-peak enhancement, higher peak enhancement, steeper wash-in slopes, and larger areas under the time–intensity curve (AUC) compared with benign lesions [13, 14, 25,18,22,23 ]. These findings are consistent with increased microvascular density, arteriovenous shunting, and disorganized angiogenesis, which are characteristic features of malignant tumor biology. Specifically, studies employing time–intensity curve analysis reported that malignant lesions reached peak enhancement more rapidly and showed higher overall contrast uptake than benign tumors, particularly pleomorphic adenomas [13, 14, 25,22 ]. Increased AUC and, in some studies, prolonged mean transit time were also observed in malignant lesions, reflecting altered microvascular perfusion and delayed contrast clearance [14, 22 ]. However, not all quantitative parameters were uniformly discriminatory across investigations, and significant overlap between malignant and highly vascular benign tumors was frequently reported. Notably, absolute cut-off values for quantitative parameters varied widely across studies, reflecting differences in CEUS acquisition protocols, region-of-interest selection, post-processing software, and contrast administration techniques [13, 14, 25,19,22,23 ]. As a result, none of the quantitative parameters could be identified as a standalone diagnostic marker with consistent thresholds applicable across cohorts. Instead, quantitative CEUS metrics were primarily reported as group-wise comparisons rather than as components of predefined diagnostic decision rules. Overall, quantitative CEUS parameters provided objective and reproducible evidence of perfusion differences between malignant and benign salivary gland lesions. Nevertheless, the lack of standardized acquisition protocols and diagnostic thresholds limited their direct applicability for pooled diagnostic accuracy analysis and supports their use as adjunctive markers within a multiparametric assessment, rather than as isolated diagnostic tests.

**Table 3:** Quantitative contrast-enhanced ultrasound (CEUS) parameters reported in studies evaluating benign and malignant focal salivary gland lesions

Quantitative CEUS parameter	Benignlesions	Malignantlesions
Time to peak (TTP)	Longer TTP	Shorter TTP
Peak enhancement (PE)	Lower peak enhancement	Higher peak enhancement
Wash-in slope	Gradual wash-in	Steeper wash-in slope
Mean transit time (MTT)	Longer MTT	Shorter MTT
Perfusion heterogeneity indices	Low intralesional variability	Increased intralesional heterogeneity

Quantitative perfusion metrics derived from time–intensity curve analysis—including time to peak (TTP), peak enhancement (PE), area under the time–intensity curve (AUC), and mean transit time (MTT)—show consistent differences between benign and malignant tumors across studies

### Diagnostic accuracy evidence and meta-analytic considerations

Among the included studies, only a minimal number provided diagnostic accuracy data suitable for malignant versus benign classification, and even these investigations differed substantially in diagnostic definitions and analytical approaches. Consequently, a formal pooled diagnostic accuracy meta-analysis was not feasible. Across the two studies reporting complete diagnostic accuracy data, CEUS sensitivity ranged from 72.1 to 80.0%, whereas specificity ranged from 78.9 to 91.7%. Zhao et al. [24] assessed differentiation between malignant tumors and focal inflammatory masses and reported a sensitivity of 80.0% and a specificity of 78.9%. Shi et al. evaluated CEUS using explicit qualitative criteria and reported a sensitivity of 72.1% and a specificity of 91.7% for malignancy, which further improved when CEUS was combined with elastography [16]. However, these studies addressed different target conditions, applied non-uniform diagnostic thresholds, and enrolled distinct lesion spectra, precluding reliable pooling of sensitivity and specificity estimates.

Large observational studies, such as Wang et al. [17], provided robust evidence on the predictive value of individual CEUS features through multivariable regression analysis but did not define a global dichotomous diagnostic threshold. As a result, 2 × 2 contingency tables could not be reconstructed, and these studies could not be incorporated into a quantitative diagnostic accuracy meta-analysis. Overall, due to the small number of eligible diagnostic accuracy studies, heterogeneity in diagnostic criteria, and absence of standardized CEUS thresholds, any pooled estimates of sensitivity or specificity would be highly unstable and potentially misleading. Accordingly, diagnostic accuracy evidence was synthesized qualitatively rather than quantitatively.

*Feature-level diagnostic accuracy*. Feature-level analysis across studies demonstrated that certain qualitative CEUS characteristics carry clinically meaningful diagnostic information. In particular, ill-defined or blurred enhancement margins consistently showed high specificity for malignancy and were associated with strong independent predictive value in multivariable models, making them useful “rule-in” features when present. In contrast, inhomogeneous enhancement was frequently observed in malignant lesions and therefore showed higher sensitivity, but it was also common in several benign or inflammatory entities, resulting in limited specificity. Consequently, inhomogeneous enhancement alone should not be used as a decisive diagnostic criterion. Wash-in and wash-out kinetics demonstrated variable diagnostic performance across studies. Differences in parameter definitions, timing thresholds, and qualitative versus quantitative assessment approaches prevented meaningful quantitative pooling. While rapid wash-in followed by early wash-out was more frequently reported in malignant lesions, this feature lacked consistency as a standalone discriminator.

## Discussion

This systematic review demonstrates that CEUS provides valuable functional information for the evaluation of focal salivary gland lesions and effectively complements conventional ultrasound by depicting tumor microvascular characteristics in real time. Both qualitative and quantitative CEUS features have been consistently associated with malignant behavior across multiple studies, supporting the biological plausibility of CEUS as a functional imaging modality in this setting. In particular, heterogeneous enhancement, ill-defined or blurred margins, non-enhancing areas suggestive of necrosis, and altered contrast kinetics reflect underlying tumor angiogenesis, vascular disorganization, and infiltrative growth patterns, which are hallmarks of malignancy [12, 13, 15–21,18 ].

Quantitative CEUS parameters further strengthen this concept by providing objective perfusion metrics. Several studies have demonstrated that malignant salivary gland tumors exhibit shorter time-to-peak enhancement, higher peak enhancement, steeper wash-in slopes, and larger areas under the time–intensity curve compared with benign lesions. These findings are consistent with increased microvascular density and abnormal vascular architecture in malignant tumors and were reproducible across studies using different analytical approaches and software platforms [13, 14, 23,22 ]. Although absolute cut-off values varied substantially across investigations, the direction of these associations was remarkably consistent, reinforcing the potential role of CEUS in preoperative risk stratification rather than in binary lesion classification.

Nevertheless, an important limitation emerging from the available evidence is the substantial overlap in CEUS appearance between malignant lesions and certain benign or inflammatory masses. Highly vascular benign tumors, such as pleomorphic adenomas and Warthin tumors, as well as focal inflammatory lesions, may demonstrate heterogeneous enhancement and rapid contrast inflow, thereby mimicking malignant perfusion patterns [13, 15, 24,18 ]. This overlap underscores the necessity of integrating CEUS findings with clinical presentation, laboratory data, and conventional ultrasound features. Accordingly, CEUS should be interpreted as a multiparametric imaging tool rather than a standalone diagnostic test capable of definitively distinguishing malignancy.

Large observational studies further illustrate both the strengths and current limitations of CEUS. For example, Wang et al. demonstrated that individual CEUS features—such as blurred enhancement margins and post-contrast lesion enlargement—were strong independent predictors of malignancy in multivariable logistic regression analysis. However, the absence of predefined global diagnostic thresholds or standardized decision rules limited the ability to derive a single dichotomous test outcome, thereby precluding inclusion in diagnostic accuracy meta-analysis [17]. Despite this limitation, such studies are essential for pattern recognition and hypothesis generation and provide a critical empirical foundation for future standardization efforts.

Finally, the limited number of high-quality diagnostic test accuracy studies highlights a significant gap in the current literature. The COVID-19 pandemic renewed interest in salivary gland pathology by highlighting the salivary glands as potential targets of SARS-CoV-2 infection. Consequently, ultrasound has gained increasing attention as a rapid, non-invasive imaging modality for the evaluation of inflammatory and structural salivary gland abnormalities [26–28]. Methodological heterogeneity in CEUS acquisition protocols, contrast agent dosing, region-of-interest selection, post-processing software, and interpretation criteria hampers direct comparison across studies and restricts robust quantitative synthesis [14, 18, 19 ]. These findings emphasize the need for prospective, multicenter investigations employing standardized CEUS protocols and clearly defined diagnostic criteria [29, 30 ]. Establishing consensus guidelines for CEUS interpretation in salivary gland imaging will be crucial to clarify its role in clinical decision-making and to determine whether CEUS can move beyond its current adjunctive function toward a more standardized diagnostic tool.

## Conclusions

CEUS is a valuable adjunct imaging modality for the assessment of focal salivary gland lesions and improves preoperative risk stratification by providing functional perfusion information. However, current evidence supports its use as a complementary tool rather than a replacement for histopathological diagnosis. Further prospective, multicenter studies with standardized protocols are required to clarify the diagnostic role of CEUS.

## Data Availability

Not applicable.
