# Correction: CEUS in the evaluation of focal salivary gland lesions: a systematic review

**DOI:** 10.1007/s40477-026-01213-0

**Published:** 2026-08-28

**Authors:** Alessio Frisone, Giuseppe Balice, Matteo Serroni, Gianmaria D’Addazio, Antonella Berardicurti, Andrea Boccatonda

**Affiliations:** 1 Department of Innovative Technologies in Medicine & Dentistry, “G. D’Annunzio” University, Via Dei Vestini 31, Chieti, Italy; 2 Department of Life Science, Health and Health Professions, Link Campus University, Macerata, Italy; 3 Department of Medicine and Science of Aging, University Gabriele d’Annunzio Chieti-Pescara, Chieti, Italy; 4 Diagnostic and Therapeutic Interventional Ultrasound Unit, IRCCS Azienda Ospedaliero-Universitaria Di Bologna, Policlinico Sant’Orsola-Malpighi, Via Massarenti N. 9, 40138 Bologna, Italy

**Correction: Journal of Ultrasound (2026)** https://doi.org/10.1007/s40477-026-01205-0

In the published article, the 4th author name has been incorrectly published. The complete correct name should read as follows.

Gianmaria D'Addazio

The original article has been corrected.

